# Manipulation of ABA Content in *Arabidopsis thaliana* Modifies Sensitivity and Oxidative Stress Response to *Dickeya dadantii* and Influences Peroxidase Activity

**DOI:** 10.3389/fpls.2017.00456

**Published:** 2017-04-03

**Authors:** Frédérique Van Gijsegem, Jacques Pédron, Oriane Patrit, Elizabeth Simond-Côte, Alessandra Maia-Grondard, Pierre Pétriacq, Raphaël Gonzalez, Lydie Blottière, Yvan Kraepiel

**Affiliations:** ^1^Interactions Plantes-Pathogènes, AgroParisTech, Institut National de la Recherche Agronomique, Université Pierre et Marie Curie – Université Paris 06Paris, France; ^2^Institut d’Ecologie et des Sciences de l’Environnement de Paris, Sorbonne Universités, Université Pierre et Marie Curie – Université Paris 06, Diderot Université Paris 07, Université Paris-Est Créteil – Université Paris 12, Centre National de la Recherche Scientifique, Institut National de la Recherche Agronomique, Institut de Recherche pour le DéveloppementParis, France; ^3^Institut Jean-Pierre Bourgin, AgroParisTech, Institut National de la Recherche AgronomiqueVersailles, France

**Keywords:** abscisic acid, peroxidases, oxidative stress, plant defense, bacterial virulence genes, *Dickeya dadantii*, *Arabidopsis thaliana*

## Abstract

The production of reactive oxygen species (ROS) is one of the first defense reactions induced in *Arabidopsis* in response to infection by the pectinolytic enterobacterium *Dickeya dadantii*. Previous results also suggest that abscisic acid (ABA) favors *D. dadantii* multiplication and spread into its hosts. Here, we confirm this hypothesis using ABA-deficient and ABA-overproducer *Arabidopsis* plants. We investigated the relationships between ABA status and ROS production in *Arabidopsis* after *D. dadantii* infection and showed that ABA status modulates the capacity of the plant to produce ROS in response to infection by decreasing the production of class III peroxidases. This mechanism takes place independently of the well-described oxidative stress related to the RBOHD NADPH oxidase. In addition to this weakening of plant defense, ABA content in the plant correlates positively with the production of some bacterial virulence factors during the first stages of infection. Both processes should enhance disease progression in presence of high ABA content. Given that infection increases transcript abundance for the ABA biosynthesis genes *AAO3* and *ABA3* and triggers ABA accumulation in leaves, we propose that *D. dadantii* manipulates ABA homeostasis as part of its virulence strategy.

## Introduction

Abscisic acid (ABA) is well-known as the major phytohormone accumulating in response to abiotic stress such as drought, salt, osmotic or cold stresses, and is involved in plant adaptation to these unfavorable conditions (Nambara and Marion-Poll, 2005). For over a decade, the implication of ABA in plant–pathogen interactions has also been clear (Ton et al., 2009; Cao et al., 2011). As ABA serves as a signal in such a diversity of plant responses to environmental factors, many recent studies have focused on its role in crosstalk between biotic and abiotic stress responses (Lee and Luan, 2012). When faced with multiple stresses, plants have to prioritize their adaptive responses and, in many cases, ABA promotes abiotic stress responses at the expense of defense reactions to pathogens (Robert-Seilaniantz et al., 2007). ABA mainly exerts this negative role in post-invasive defense, as exemplified by the inhibition of callose deposition and PAMP-induced gene expression elicited by the bacterial pathogen *Pseudomonas syringae* pv. *tomato* (de Torres Zabala et al., 2009). Nevertheless, ABA also promotes stomatal pre-invasive resistance to foliar bacterial pathogens (Melotto et al., 2006) and stimulates callose accumulation in papillae in response to the fungal necrotrophic pathogen *Leptosphaeria maculens* (Ton et al., 2009; Cao et al., 2011). Thus, ABA exhibits contrasted roles in plant defense depending on infection phase and pathogen lifestyle. This highlights the importance of studying each interaction and its kinetics individually.

Abscisic acid modulates plant defense to pathogens through direct and indirect mechanisms. In *Arabidopsis*, ABA directly decreases phytoalexins, lignin, and salicylic acid (SA) levels by inhibiting expression of many genes important for phenylpropanoid biosynthesis, as *PAL1* encoding a phenylalanine ammonia lyase (Mohr and Cahill, 2007). Recent studies highlighted the major involvement of ABA in the regulation network of plant defense. It induces the accumulation of HLS1, an histone acetyltransferase that regulates epigenetically defense responses (Liao et al., 2016), enhances expression of defense genes through the down-regulation of many miRNAs (Cheng et al., 2016) and its effects on cell wall composition and structure influence resistance to pathogens (Curvers et al., 2010; Sánchez-Vallet et al., 2012). All these data would explain the negative impact of ABA on pathogen defense in some pathosystems (Asselbergh et al., 2008b). ABA may also act through mutualistic antagonism with SA and jasmonic acid (JA)/ethylene (ET) signaling pathways. This seems to be a major mechanism leading to ABA-induced susceptibility to many pathogens (Robert-Seilaniantz et al., 2007; Derksen et al., 2013). Interestingly, modulation of ABA levels has also been described as part of the virulence strategy of plant pathogens. Indeed *P. syringae* pv. *tomato* DC3000 induces ABA accumulation and production of ABA signaling components in *Arabidopsis*, thus favoring bacterial multiplication and disease progression (de Torres-Zabala et al., 2007). Moreover, some pathogenic fungi, such as *Botrytis cinerea* and *Magnaporthe grisea*, directly produce ABA, thereby improving the efficiency of the infection process (Cao et al., 2011).

Production of reactive oxygen species (ROS) is another early general response of plants to pathogen attacks. It has both an anti-microbial effect, including cell-wall reinforcement through protein crosslinking, and a signaling role in SA response, systemic acquired resistance (SAR) establishment and hypersensitive response (Apel and Hirt, 2004). Perception of pathogen associated molecular patterns (PAMPs), or bacterial effectors, induces a transduction cascade including ion fluxes and protein kinase activation that in turn enhances ROS production. Plasma membrane-bound NADPH oxidases (RBOH) play a major role in this oxidative burst but apoplastic peroxidases and some apoplast-, chloroplast-, peroxisome-, or mitochondrion-located oxidases have also been implicated in ROS production during plant–pathogen interaction (Bolwell and Wojtaszek, 1997; Apel and Hirt, 2004). Interestingly, different mechanisms link ABA levels and redox homeostasis in *Arabidopsis* seedlings. First, ABA induces the transcription of genes involved in the ROS-scavenging ascorbate-glutathione cycle (Ghassemian et al., 2008). Second, ABA biosynthesis from the carotenoid pigment violaxanthin indirectly lowers the use of the antioxidant ascorbate and decreases ROS levels. Indeed, de-epoxidation of violaxanthin into zeaxanthin in the xanthophyll cycle requires the oxidation of ascorbate into de-hydro ascorbate. This metabolic process would explain why ABA deficient mutants have lower ascorbate contents and accumulate ROS (Ton et al., 2009). Grant and Jones (2009) proposed another interesting molecular mechanism to explain the inhibition of ROS accumulation in response to ABA. ABA may induce the expression of genes encoding ROS-detoxifying enzymes, through the stabilization of DELLA proteins, which are key regulators in integrating hormone and environmental signals (Achard et al., 2008; Yang et al., 2012). Nonetheless, the inverse effect of ABA has also been described with ABA-overproducer transgenic tobacco plants accumulating ROS in mesophyll cells (Zhang et al., 2009).

*Dickeya dadantii* (formerly *Erwinia chrysanthemi*) is a pectinolytic enterobacterium responsible for soft rot on a wide host range of plants including the model plants tomato and *Arabidopsis*. Soft rot is mainly characterized by disintegration of plant tissues that is due to the production and secretion of cell wall degrading enzymes. These are pectinases and a cellulase secreted by the Out type II secretion system (T2SS) and proteases secreted by the Prt type I secretion system (T1SS). The injection of effectors into host cells by the type III secretion system (T3SS) plays only a minor role in *D. dadantii* virulence, in contrast to type III dependent bacteria such as *P. syringae* or *Xanthomonas campestris*. Before appearance of the ultimate maceration symptom, other virulence factors are required for infection and colonization of plant tissues. Adherence, aggregate formation and motility onto the leaf surface are needed for penetration of the leaf surface. Once inside the apoplast, bacteria encounter environmental stresses such as low iron availability, low apoplastic pH, microaerophilic conditions and the presence of antimicrobial peptides. Perception of and adaptation to these conditions allow bacterial density reaches a sufficient threshold for growth into plant tissues and the production of cell wall degrading enzymes. Of all the stresses bacteria have to cope with, it should be noted that the high concentrations of ROS produced by plants are the first line of defense. Bacteria achieve ROS detoxification with antioxidant enzymes such as superoxide dismutase and through the production of the ROS scavenger blue pigment indigoidine. Bacterial mutants impaired in each of these antioxidant processes exhibit a dramatic loss of virulence demonstrating their major role in infection (see Charkowski et al., 2012; Reverchon and Nasser, 2013 for reviews).

Analysis of host plant responses to *D. dadantii* infection focused mainly on tomato and *Arabidopsis* defense reactions. Induction of JA and SA pathways (Fagard et al., 2007), iron transport and storage (Dellagi et al., 2005, 2009) and ROS production associated with cell wall protein cross-linking (Fagard et al., 2007; Asselbergh et al., 2008a; Kraepiel et al., 2011) have been demonstrated. We have also shown that the bacterium induces necrosis around the maceration zone in *Arabidopsis* leaves, which can stop disease progression (Kraepiel et al., 2011), highlighting the importance of bacterial spread into healthy tissues before the symptoms appear. Phenotypic analyses of plant mutants have, however, shown that in most cases disruption of a single defense reaction has only a weak, if any, effect on disease development. Two notable exceptions are illustrated by the much higher susceptibility of JA-related and *AtRbohD Arabidopsis* mutants (Fagard et al., 2007). Notwithstanding, this broad host-range bacterium is able to bypass the multifactorial defense process induced after infection.

We have previously shown that ABA-deficient tomato mutants exhibit a resistant phenotype (Asselbergh et al., 2008a) and that ABA-hypersensitive *Arabidopsis* mutants, identified from a hot-leaf phenotype, are highly susceptible to *D. dadantii* (Plessis et al., 2011). This revealed that ABA-related modifications of plant physiology strongly influence bacterium multiplication and spread into the leaves of both hosts. In tomato, ABA down-regulates the apoplastic peroxidase activity that generates ROS. This correlates with an ABA-induced susceptibility of tomato plants to *D. dadantii* (Asselbergh et al., 2008a). To date, the effect of ABA on peroxidases and ROS production has not been investigated during infection of *Arabidopsis* by *D. dadantii*, a pathosystem where the efficient ROS producing enzyme involved in plant defense has been identified as the RBOHD NADPH oxidase (Fagard et al., 2007). In order to investigate the relationships between ABA-related responses and oxidative stress in detail during *Arabidopsis* infection by *D. dadantii*, we have studied the implication of ABA during the first stages of *Arabidopsis* infection. We analyzed ABA status and the regulation of its biosynthesis genes. We have investigated the relationships between plant hormonal status and the bacterial expression of virulence genes using ABA-deficient and ABA-overproducer plants. Finally, using a double mutant deficient for both ABA biosynthesis and RBOHD-related ROS production, our study indicates that, in *Arabidopsis* as in tomato, ABA modulates oxygen peroxide production *via* the control of peroxidase activity level. These data highlight the diversity of ROS sources during plant–bacteria interactions.

## Materials and Methods

### Plant Material and Bacterial Strains

All *Arabidopsis* mutants used in this study are in the Col-0 background. The ABA-deficient mutant *aba3-1* (Léon-Kloosterziel et al., 1996) and the ABA-overproducer transgenic plant *35S::NCED6* (Lefebvre et al., 2006) were kindly provided by Annie Marion-Poll (INRA, Versailles, France) and the *AtrbohD* mutant impaired in a NADPH oxidase-encoding gene (Torres et al., 2002) was provided by Mathilde Fagard (INRA, Versailles, France). Plants were grown under short day conditions at 24°C/19°C (8 h day/16 h night). The seeds were sown by batch in soil and grown for 3 weeks. Seedlings were then transplanted, three plants per pot, to 7 cm × 7 cm pots and grown for a further 3 weeks. The 6 week-old plants were incubated in small transparent containers with abundant watering to maintain 100% humidity 16 h before inoculation and throughout the infection process.

*Dickeya dadantii* strains used in this study derived from the 3937 wild type (WT) strain and were previously described (Kraepiel et al., 2011). The secretion mutants *prtE*, *outC*, and *hrcC* are respectively impaired in the type I, type II, and type III protein secretion systems. All strains were grown at 30°C in Luria-Bertani LB medium. For plant inoculation, an aliquot of bacterial stock was streaked onto LB solidified medium (1.5% Difco agar) and grown for 2 days. A single colony of each strain was then used to inoculate 5 mL liquid cultures. After 8 h of growth, 100 μL of these cultures were plated on agar medium and incubated overnight. The bacteria were re-suspended in the inoculation buffer (50 mM KPO_4_, pH 7) to an OD_600_ of 0.1, corresponding to a concentration of 10^8^ cfu ml^-1^ and then diluted to the indicated concentrations.

### Infection Methods and Symptom Scoring

Symptom progression analysis was performed as described in Lebeau et al. (2008). Bacteria were diluted in the inoculation buffer to a concentration of 10^4^ bacteria per ml and inoculation was performed by wounding one leaf per plant with a needle and depositing a 5 μl droplet of this bacterial suspension (i.e., around 50 bacteria) onto the wound. This method allows the scoring of individual symptom progression and wounding is necessary to obtain high rates of synchronized disease initiation. Between 20 and 30 plants were tested for each assay. Progression of symptoms was scored for 7 days using the following four stage symptom scale: stage 0, no symptom; stage 1, maceration around the bacterial droplet; stage 2, maceration spreading on the leaf limb; stage 3, maceration of the whole limb. Significance of the observed differences was established using the Fisher’s exact test (two sided *p*-value).

For RNA isolation, 6 week-old *Arabidopsis* plants were infected by rapid immersion in a bacterial suspension (5.10^7^ cfu ml^-1^) in inoculation buffer containing 0.01% (v/v) of the Silwet L-77 surfactant (van Meeuwen Chemicals BV, Weesp, The Netherlands). Aerial plant tissues were collected at different time points post-inoculation and ground in liquid nitrogen to a fine powder. This infection procedure, close to a natural leaf infection by splashing, was compatible with short studies of infection rate and avoided wound-related transcriptional responses (Kraepiel et al., 2011).

The infection by immersion described above did not allow us to detect any hormonal changes during disease progression (data not shown). In order to amplify the plant hormonal response to infection, ABA content was analyzed after syringe-infiltration with about 20 μl of a bacterial suspension (10^5^ or 10^7^ cfu ml^-1^) of all developed leaves for each plant. This classical infection method (Dellagi et al., 2005; Fagard et al., 2007) bypasses the bacterial penetration step of leaf infection and greatly increases the number of plant cells directly in contact with bacteria.

### RNA Extraction and Analysis

Total RNAs were purified as described in Lebeau et al. (2008). Briefly, RNAs were extracted in a guanidium isothiocyanate extraction buffer and pelleted by centrifugation on a cesium chloride cushion. Pellets were washed twice with 70% RNAse-free ethanol and dissolved in RNAse-free water. RNA samples were treated with RNAse-free DNAse I (Invitrogen) to remove any DNA contamination. First-strand cDNAs were then synthesized from 2 μg of total RNA using M-MLV reverse transcriptase and oligo(dT20) or random primers for plant and bacterial genes analysis respectively, following the manufacturer’s instructions (Invitrogen).

For quantitative Real-Time PCR analysis, cDNAs were amplified using Maxima^®^ SYBR Green/ROX qPCR Master Mix (Fermentas) according to manufacturer’s license in an Applied Biosystems 7300 Real Time PCR System using the following conditions: 10 min at 95°C followed by 40 amplification cycles each consisting of 15 s at 95°C and 60 s at 60°C. Results were analyzed with the Applied Biosystems Sequence Detection Software v1.3.1.

To normalize the expression data, the *Arabidopsis ß-6 TUBULIN* gene (*TUB6)* and the *D. dadantii* ß subunit of RNA polymerase-encoding gene (*RpoB*) were used as internal constitutive controls. The comparative quantitation method (ΔΔCt) was used to contrast the different conditions (Livak and Schmittgen, 2001). Ct values quantify the number of PCR cycles necessary to amplify a template to a chosen threshold concentration, ΔCt values quantify the difference in Ct values between a test and a control gene for a given sample, and ΔΔCt values are used for the comparison between two samples. ΔΔCt values were transformed to absolute values with 2^-ΔΔCt^ to obtain relative transcript levels. References for relative transcript levels were set to one. All primers used for transcript quantification are listed in **Table 1**.

**Table 1:** Primers used for quantitative real-time RT-PCR.

Name	AGI ID	Forward	Reverse
**Primers for plant gene expression studies**			
*AAO3*	AT2G27150	AAATCTCCACACCCACTTCG	CCCCATTAACTGCAAACTCC
*ABA3*	AT1G16540	AAGAGCAAGCGGTGGATG	GCCAAGCCCAGTAGGATAAC
*TUB6*	AT5G12250	TGGATCATGAGTGAGTGAAAAGA	ACCGACCAAACGAAAAGAAG

**Name**	**ASAP ID**	**Forward**	**Reverse**

**Primers for bacterial gene expression studies**			
*indC*	ABF-0016081	TCGCTCTGGCTCGTTATCTT	GGCGTCATCCAGGTCATTAT
*pelI*	ABF-0014586	TGGCGACTATCAGTGGTCTG	ACAGTTGGTGGTGTCCCATT
*prtC*	ABF-0020371	TGAGCTTTGTGCAGGATCAG	CCAGGAAGTCTACCGAGCTG
*rpoB*	ABF-0014902	GAATTGGTTACCTGCCGTAGCA	AACGTCCATGTAGTCAACCTGATC

*Primer efficiency (E) was calculated from standard curve slope according to E = (10 ^(-*1/slope*)^ – 1) × 100. Primers were selected in a range of 100 ± 5% efficiency.*

### ABA Content Measurement

The mature leaves of nine plants per time point were infiltrated with a bacterial suspension or with the inoculation buffer. After harvest, leaves were directly frozen in liquid nitrogen and freeze-dried. Pooled dried leaves were ground in a ball mill (Mixer Mill MM200, Retsch) and 100 mg of the powder obtained used for ABA content determination as described by Plessis et al. (2011). Briefly, 2 mL of extraction solvent (acetone, water, acetic acid, 80/19/1, v/v/v) containing 30 ng of ^2^H-ABA (-)-5,8′,8′,8′-d4 ABA purchased from Irina Zaharia (Plant Biotechnology Institute – National Research Council, Canada) as internal standard were added to the leaf powder and carefully mixed. The supernatant was recovered by centrifugation and the pellet was rinsed with 1 mL of extraction solvent. The extraction solvent was evaporated and the residue was resuspended in 0.5 mL of HPLC solvent (acetonitrile, water, acetic acid, 50/50/0.05, v/v/v). ABA was quantified using LC-ESI-MS-MS system (Quattro LC, Waters^1^) in positive ionization and multiple reaction monitoring mode. The differences in ABA content between the different samples were assessed using the non-parametric Kruskal–Wallis analysis of variance for each time point. A *p*-value of ≤0.05 was considered statistically significant.

### H_2_O_2_ Detection in Leaves

Detection of H_2_O_2_ using 3,3′-diaminobenzidine (DAB, Sigma) staining was performed as described by Torres et al. (2002). Twenty to 30 individual plants for each genotype were inoculated on a single leaf with 5 μL of a 5.10^7^ cfu ml^-1^ bacterial suspension by the wounding method. To compare the different genotypes at the same stage of disease, we selected 12 leaves of each genotype exhibiting a stage 1 symptom (maceration around the bacterial droplet) for staining 24 h post-inoculation.

### *In vitro* Class III Peroxidase Activity Assays

The *in vitro* peroxidase activity assay was adapted from Cesarino et al. (2012). Inoculation was performed in the same conditions as described for H_2_O_2_ detection and 10 leaves of each genotype exhibiting a stage 1 symptom were harvested and frozen in liquid nitrogen 24 h post-inoculation for enzymatic activity assay. Pooled frozen leaves were grounded to a fine powder using a ball mill and soluble proteins were extracted from tissue, corresponding to about 300 mg of fresh weight, in 1 mL of extraction buffer [100 mM KPO_4_ pH 7.8; 0.5% v/v Triton X-100; 2% w/v poly(vinylpolypyrrolidone)] by vortexing. Extracts were centrifuged (30 min, 20000 × *g*, 4°C), supernatants were gel-filtrated though Sephadex G25 columns (PD miditrap G25, GE Healthcare) and eluted with 1.5 mL of 100 mM KPO_4_ buffer (pH 7.8). The extracts were then concentrated to 50–100 μL using Amicon ultra-4 Centrifugal Filter units (Ultracel-10 membrane) and protein concentrations were determined using the Bio-Rad protein assay. The peroxidase activity assay (200 μL) contained 10 μg proteins, 50 mM Na acetate (pH 5), 8.26 mM guaiacol and 0.03% (v/v) H_2_O_2_. OD_470__mn_ was followed during 15 min (25°C) in a microplate reader Spectramax 190 (Molecular Devices). Enzyme activity was calculated from plot gradients as the production rate of tetraguaiacol (ε = 26.6 mM^-1^ cm^-1^) expressed as μmol min^-1^ mg^-1^ total proteins. In all experiments, flat plots were obtained in the absence of H_2_O_2_ or protein extract and confirmed the specificity of the assay. The differences in peroxidase activity between all genotypes were assessed by the non-parametric Kruskal–Wallis analysis of variance. The differences that resulted when comparing two genotypes were assessed by the Wilcoxon test. A *p*-value of ≤0.05 was considered statistically significant.

## Results

### ABA Enhances Susceptibility of *Arabidopsis thaliana* to *Dickeya dadantii*

**FIGURE 1:**
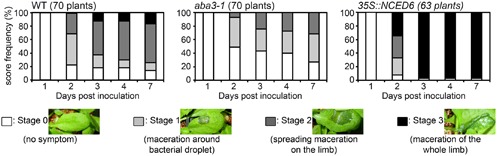
**Rates of progression of soft rot symptoms in *aba3-1* and *35S::NCED6* plants.** A single leaf per plant was inoculated with a 5 μL drop of 10^4^ cfu ml^-1^ bacterial wild type (WT) strain (3937) suspension onto a needle wound. The number of plants scored for each genotype is indicated and corresponds to the sum of three independent experiments. Significance of the observed differences was established using the Fisher’s exact test (two sided *p*-value) (see Supplementary Table S1 for statistical values).

We have previously shown that ABA hypersensitive mutants of *Arabidopsis* exhibit an increased susceptibility to *D. dadantii* indicating a role for ABA in the *Arabidopsis* resistance response (Plessis et al., 2011). To confirm this hypothesis, we followed symptom occurrence and disease progression after inoculation of *D. dadantii* WT strain (3937) on the WT genotype Col-0, the ABA-deficient mutant *aba3-1* and the ABA-overproducing *35S::NCED6* transgenic plants. Symptoms were scored each day over the first 4 days and then 7 days post-inoculation (dpi) (**Figure 1** and Supplementary Table S1). As early as 2 dpi, the *aba3-1* mutant had less leaves with symptoms and maceration was not as widespread compared to the WT, whereas the ABA-overproducing genotype had very few healthy leaves and more severe symptoms. Similarly, at the end of the infection process (7 dpi), all maceration had stopped on the ABA-deficient mutant resulting in less severe symptoms than on WT leaves, while ABA-overproducing transgenic plants exhibited complete maceration of most inoculated leaves (**Figure 1**). These results demonstrate unambiguously the involvement of the phytohormone ABA in both disease initiation and progression during *D. dadantii* infection of *Arabidopsis* leaves. This observation led us to hypothesize that *D. dadantii* may manipulate ABA-content as part of its virulence strategy.

### *Dickeya dadantii* Induces ABA Production in *Arabidopsis* Leaves and Transcription of the Biosynthesis Genes AAO3 and ABA3

**FIGURE 2:**
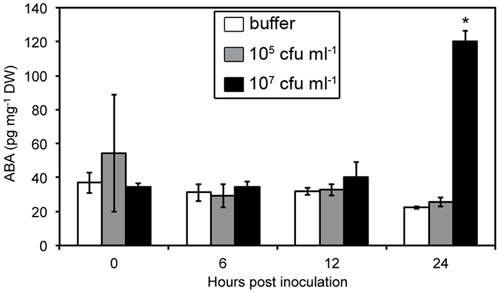
**Abscisic acid (ABA) content in WT Col-0 leaves over the first 24 h following leaf infiltration with *Dickeya dadantii* wild strain (3937) suspensions at the indicated concentrations.** Results presented correspond to the mean and standard deviation of three replicates of nine plant pools. Asterisks indicate a significant difference associated with the global multigroup comparison (Kruskal–Wallis test) at the time point indicated.

We measured ABA levels in *Arabidopsis* leaves over 24 h after infiltration with a bacterial suspension containing 10^5^ or 10^7^ bacteria per mL (**Figure 2**). The lowest concentration led to very little maceration 24 h post-inoculation (hpi) while we observed clear symptoms 24 hpi using the highest concentration. We observed a strong increase in ABA levels in infected leaves, but only after 24 hpi with the highest inoculum. Increases in ABA-content were, therefore, only detected once bacterial cell wall degrading enzymes were secreted and the first maceration symptoms observed.

**FIGURE 3:**
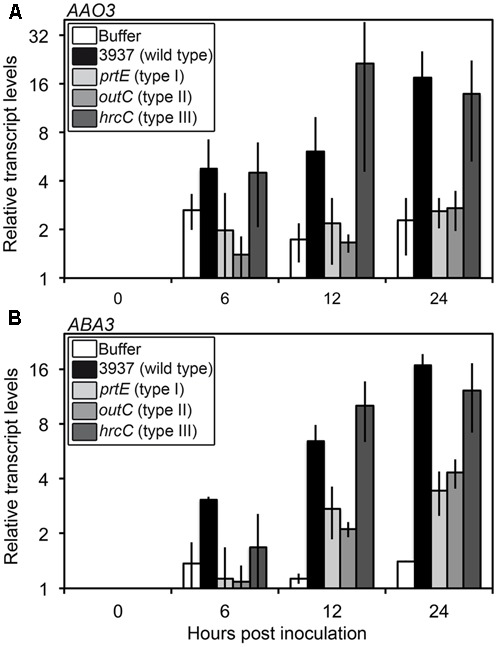
**Expression of genes involved in the last step of ABA biosynthesis in WT Col-0 infected with *D. dadantii* WT or protein secretion mutant strains.** Six-week-old plants were inoculated by immersion into 5.10^7^ cfu ml^-1^ bacterial suspensions or phosphate buffer as control. Bacterial strains used were the 3937 WT strain, the *prtE* type I secretion system mutant, the *outC* type II secretion system mutant and the *hrcC* type III secretion system mutant. Four to six plant rosettes were harvested at the indicated time points and expression of genes encoding the abscisic aldehyde oxidase AAO3 **(A)** and the molybdenum cofactor sulfurase ABA3 **(B)** was analyzed by quantitative real time RT-PCR using *BETA-6 TUBULIN* as a constitutive gene. Relative transcript levels were expressed according to the reference condition (0 hpi) set to 1 for each bacterial strain. Bars correspond to the mean of two independent replicates and error bars indicate the spread between the two values. Statistical analysis was performed comparing the kinetics to the buffer inoculation with a linear mixed-effects model using strain*hpi as a fixed effect and the bacterial strains as the random effect. Expression kinetics after infection by 3937 and *hrcC* strains were significantly different from that obtained with buffer inoculation (*AAO3*: *p*-value = 0.0116 and 0.0239 respectively; *ABA3*: *p*-value = 0.0004 and 0.0006 respectively) whereas differences between kinetics after inoculations by buffer and *prtE* or *outC* were not significant or only weakly significant (*AAO3*: *p*-value = 0.5711 and 0.5504 respectively; *ABA3: p*-value = 0.0830 and 0.0411 respectively) (see Supplementary Table S2 for complete statistical data).

Transcriptional activation of ABA biosynthesis genes, mainly 9-*cis*-epoxycarotenoid dioxygenase (*NCED2*, *NCED3*, *NCED5*), abscisic aldehyde oxidase (*AAO3*) and molybdenum cofactor sulfurase (*ABA3*) encoding genes, contribute to increases in ABA levels in response to stress (Nambara and Marion-Poll, 2005). Transcript abundance was determined for these five genes during the first 24 hpi. Of these, only *AAO3* and *ABA3* exhibited detectable increased transcript levels in response to infection by the WT bacterial strain (3937) as compared to the buffer treatment (**Figure 3** and Supplementary Table S2). For both genes, differential transcript accumulation started as early as 6 hpi and increased until 24 hpi.

This increase in *AAO3* and *ABA3* transcripts levels was detected very early during the infection process, before ABA content increased and symptoms appeared, and coincided with the induction of bacterial virulence factor encoding genes, that takes place around 12 hpi (Lebeau et al., 2008). This suggests that specific bacterial signals could induce ABA accumulation through transcriptional activation of its biosynthesis. We tested this hypothesis by analyzing the expression of ABA biosynthesis genes after inoculation of the *prtE*, *outC*, and *hrcC* bacterial mutants impaired in the secretion of proteases, pectinases and cellulase, and type III effectors respectively (**Figure 3**). The *hrcC* mutant induced the *AAO3* and *ABA3* genes in a manner comparable to the WT strain. Interestingly, the *prtE* and *outC* mutants appeared unable to induce *AAO3* expression, since the corresponding expression kinetics are not significantly different from that obtained with the buffer inoculation (Supplementary Table S2), and triggered a weak increase in *ABA3* transcripts level compared to the WT strain. Indeed, *ABA3* expression kinetics obtained after infection by *prtE* and *outC* are not significantly or weakly significantly different from that obtained after buffer inoculation (*p*-value = 0.0830 and 0.0411, respectively) in contrast to the highly significant differences between the WT strain (*p*-value = 0.0004) or the *hrcC* mutant (*p*-value = 0.0006) and the buffer (**Figure 3** and Supplementary Table S2). These data indicate that infecting bacteria must secrete both proteases and cell wall degrading enzymes in order to induce ABA biosynthesis gene expression and thereby induce ABA accumulation.

### ABA Status in Plant Modulates the Production of Virulence Factors in Bacteria

**FIGURE 4:**
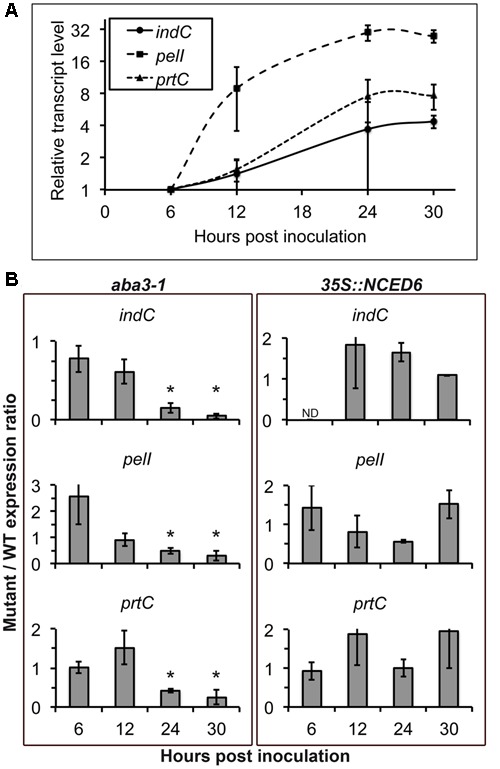
**Effect of plant ABA content on bacterial virulence gene expression.** The 3937 bacterial WT strain was inoculated by immersion of WT Col-0, the *aba3-1* mutant and *35S::NCED6* plants. Four to six plant rosettes were harvested at the indicated time points and expression of bacterial genes encoding the indigoïdin synthase IndC, the pectinase PelI and the protease PrtC were analyzed by quantitative real time RT-PCR using *RpoB* as a constitutive gene. **(A)** Relative transcript levels after infection of WT Col-0 plants were expressed according to the 6 hpi time point set to 1 for each bacterial gene (Mhedbi-Hajri et al., 2011). Each point corresponds to the mean of three independent replicates ± standard error. **(B)** Transcript accumulation was quantified after infection of *aba3-1* and *35S::NCED6* plants and was expressed as the ratio of mutant or transgenic expression to WT (mutant/WT expression ratio), comparing levels measured on genotypes at the same time point after inoculation. Histograms correspond to the mean of 3 replicates and the error bars represent standard deviation. Fold changes comprised between 0.5 and 2 were not considered significant. ND, not detectable. Asterisks indicate significant differences (*p* < 0.05) between the mutant and the WT (Wilcoxon test).

The positive correlation between symptom severity and ABA contents could relate to a modulation of plant defense by ABA and/or to the influence of the physiological status of the plant tissues on bacterial virulence. To test this latter hypothesis, we analyzed the expression of some bacterial virulence factors encoding genes in the WT bacterial strain inoculated on the WT plant Col-0, the ABA-deficient *aba3-1* mutant and the ABA-overproducing *35S::NCED6* transgenic plant. The expression of PelA, PelC, and PelD pectinase-encoding genes was induced about 10 to 30-fold during the infection process (Mhedbi-Hajri et al., 2011), and showed similar profiles after infection of the different plant genotypes (Supplementary Figure S1). In contrast, the approximately fivefold increase in the accumulation of *indC* transcripts, involved in the production of the ROS-scavenger indigoidine, observed during the infection of WT (**Figure 4A**) was abolished in the *aba3-1* mutant at 24 and 30 hpi (**Figure 4B**). To a lesser extent, the 30- and 8-fold increase in expression of *pelI* and *prtC* genes, encoding the PelI pectinase and the PrtC protease respectively, observed after Col-0 infection (**Figure 4A**) were 2- to 5-fold weaker 24 h and 30 h after infection of the *aba3-1* mutant (**Figure 4B**). Accumulation of ABA in *Arabidopsis* plants infected with *D. dadantii* is thus an important metabolic modification that is required to fully induce the expression of some bacterial virulence genes. No reproducible increase (i.e., >2) was observed, however, in the expression of virulence factor encoding genes during infection of ABA-overproducing *35S::NCED6* transgenic plants compared to WT (**Figure 4**), despite *D. dadantii* provoking more severe disease symptoms in this genotype.

### ABA Inhibits Infection-Induced Hydrogen Peroxide Accumulation and Decreases Peroxidase Activity in Leaves

**FIGURE 5:**
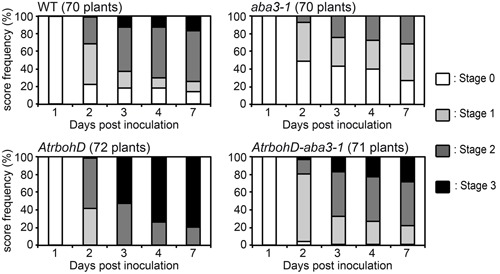
**Rates of progression of soft rot symptoms in ATRBOHD NADPH oxidase deficient genotypes.** A single leaf per plant was inoculated with a 5 μL drop of 10^4^ cfu ml^-1^ bacterial WT strain (3937) suspension onto a needle wound. The number of plants scored for each genotype is indicated and corresponds to the sum of three independent experiments. Significance of the observed differences was established using the Fisher’s exact test (two sided *p*-value) (see Supplementary Table S1 for statistical values).

The ABA status dependent expression of the oxidative stress-related bacterial *indC* gene coupled with previous work highlighting the importance of H_2_O_2_ production in *Arabidopsis-D. dadantii* interactions (Fagard et al., 2007; Kraepiel et al., 2011) might be related to the regulation of ROS production by ABA in different physiological contexts (Apel and Hirt, 2004). This led us to analyze the involvement of ROS in the ABA-related susceptibility of *Arabidopsis* to *D. dadantii*. To achieve this, we generated an *AtrbohD-aba3-1* double mutant deficient for both oxidative stress triggered by *D. dadantii* infection and ABA production. We compared symptom progression after *D. dadantii* inoculation in this genotype to that of the corresponding single mutants (**Figure 5** and Supplementary Table S1). In accordance to previously published results (Fagard et al., 2007), the *AtrbohD* mutant exhibited an extreme susceptibility to the bacterium, almost comparable to that of the ABA overproducer, with 80% of leaves entirely macerated at 7 dpi. From 2 to 7 dpi, maceration symptoms at stages ≥2 observed on the double mutant were intermediate between the resistance of the *aba3-1* mutant and the dramatic susceptibility of the *AtrbohD* genotype, and were finally not significantly different from that observed for WT (**Figure 5** and Supplementary Table S1). These data show that there is no epistasis of one gene to the other in the maceration spreading phenotype and led us to exclude the hypothesis of an involvement of AtRBOHD-related ROS production in this ABA-modulated susceptibility. We did not, however, detect any significant difference between the double mutant and the *AtrbohD* single mutant in the initiation of maceration (stage 0, 2 dpi). This latter result suggests that AtRBOHD activity plays the major role very early during infection.

**FIGURE 6:**
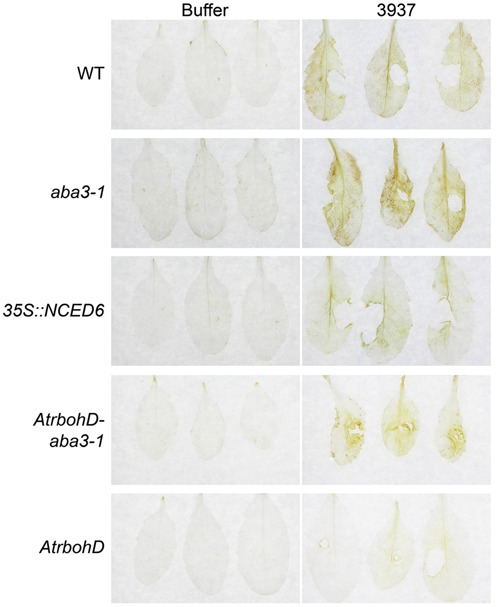
**Effects of ABA content and *AtrbohD* mutation on oxidative stress in infected leaves.** H_2_O_2_ accumulation was analyzed by Diaminobenzidine-staining of WT Col-0, *aba3-1*, *35S::NCED6*, *AtrbohD* and *AtrbohD-aba3-1* leaves 24 hpi. Infection was performed by depositing 5 μL of a 5.10^7^ cfu ml^-1^ WT bacterial strain (3937) suspension onto a needle-wound. Stage 1 symptoms (see Materials and Methods and **Figure 1**) were selected for staining comparison. Buffer inoculation was used as control. The experiment was performed three times with similar results and three representative leaves are presented from at least 12 analyzed.

Since AtRBOHD was described as the major source of ROS accumulation during *Arabidopsis-D. dadantii* interaction (Fagard et al., 2007), we examined H_2_O_2_ production in the *AtrbohD-aba3-1* double mutant during infection. Diaminobenzidine (DAB)- staining of H_2_O_2_ accumulation in leaves exhibiting stage 1 symptoms was compared between WT, the *aba3-1* and *AtrbohD* single mutants, the *aba3-1- AtrbohD* double mutant and the *35S::NCED6* transgenic plants 24 h after inoculation with the WT bacterial strain 3937 (**Figure 6**). Given the heterogeneity of disease initiation (**Figure 1**), we used leaves exhibiting symptoms around the infection site at 24 hpi as the earlier time point usable to compare leaves dealing with equivalent bacterial populations. Whereas staining was never observed on buffer inoculated leaves, bacteria induced a detectable diffused H_2_O_2_ production in the whole limb of infected WT leaves. This oxidative stress was enhanced in the *aba3-1* mutant demonstrating a down-regulation of H_2_O_2_ production by ABA. In accordance with this, the ABA-overproducing *35S::NCED6* transgenic plant exhibited a decreased DAB-staining, compared to the WT, essentially restricted to the area along the major vein. Furthermore, no DAB-staining was observed on *AtrbohD* leaves confirming that the AtRBOHD NADPH oxidase represents the main source of ROS in response to *D. dadantii* infection, when ABA is normally produced. Unexpectedly however, we detected a DAB-staining, mainly localized around the infection site, on the *AtrbohD-aba3-1* double mutant (**Figure 6**). This revealed the existence of an infection-driven AtRBOHD-independent source of H_2_O_2_ that is down-regulated by ABA.

**FIGURE 7:**
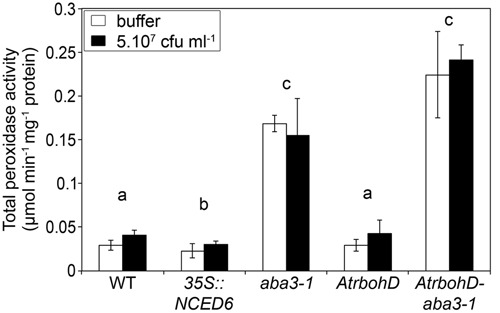
**Effects of ABA content and *AtrbohD* mutation on total peroxidase activity in *Arabidopsis* leaves.** Inoculation was performed by depositing 5 μL of a 5.10^7^ cfu ml^-1^ WT bacterial strain (3937) suspension or buffer onto a needle-wound. Leaves with stage 1 symptoms (see Materials and Methods and **Figure 1**) were selected for comparison of enzymatic activity. Proteins were extracted, gel-filtrated and concentrated prior to spectrophotometric measurement of total class III peroxidase activity, using guaiacol as substrate. Data represent the mean of 3 replicates ± standard deviation. Non-infected leaves were also analyzed twice for each genotype and the results were equivalent to those of buffer inoculations. The *p*-value associated with the global multigroup comparison, including the five genotypes (Kruskal–Wallis test) is *p* = 0.0005 and letters indicate differences (*p* < 0.05) between genotypes (Wilcoxon test).

The class III peroxidases enzymes are involved in H_2_O_2_ production in response to biotic stresses (Almagro et al., 2009). We investigated their possible role in the H_2_O_2_ producing mechanism revealed in the *AtrabohD-aba3-1* double mutant. Using guaiacol as a substrate, we analyzed *in vitro* the total class III peroxidase activity in the five previously studied genotypes 24 h after inoculation either with the inoculation buffer or with the WT bacterial strain 3937 (**Figure 7**). For all genotypes used in this study, the peroxidase activity was comparable between the bacteria-infected plants and the buffer inoculated controls. Moreover, we did not observe any difference in peroxidase activity between the WT and the *AtrbohD* mutant. We did, however, observe significantly more peroxidase activity in the *aba3-1* mutant compared to WT (*p* < 0.01) (**Figure 7**) in accordance with the data obtained in tomato by Asselbergh et al. (2008a). The *AtrbohD-aba3-1* double mutant also exhibited a high peroxidase activity similar to that we observed in the *aba3-1* mutant (*p* = 0.6). This latter result indicates that the AtRBOHD activity does not interfere with the ABA-related control of peroxidase activity revealed in the *aba3-1* mutant (**Figure 7**). In addition, peroxidase activity was slightly, but significantly, reduced in the *35S::NCED6* ABA-overproducing plants compared to WT (*p* < 0.05). It appears that the high peroxidase activity in *aba3-1*-containing genotypes (**Figure 7**) is correlated with an increase in H_2_O_2_ production in these genotypes in response to infection (**Figure 6**). Nonetheless, DAB-staining was specific to infected leaves while high peroxidase activities measured *in vitro* in ABA-deficient genotypes were also present in uninfected leaves. This indicates that bacterial infection does not induce peroxidase activity, but would trigger H_2_O_2_ production from the permanently higher peroxidase levels present in the context of low ABA contents.

## Discussion

The virulence determinants of *D. dadantii* have been extensively studied combining mutant analyses and molecular approaches (Charkowski et al., 2012; Reverchon and Nasser, 2013). In contrast, few studies have examined how manipulation of plant physiology by *D. dadantii* influences the interaction outcome. For example, siderophores secreted into leaf tissues modify the SA and JA-related defense responses (Dellagi et al., 2009; Aznar et al., 2014) and unknown bacterial signals lower the oxidative stress (Kraepiel et al., 2011). This study focuses on the role of ABA in the interaction between *D. dadantii* and *Arabidopsis*. We show that type I and type II bacterial protein secretion systems are needed to activate the expression of genes involved in ABA biosynthesis during infection and that production of some bacterial virulence factors depends on the ABA status in plant tissues. Thus, modulation of ABA status in plants influences bacterial aggressiveness. Furthermore, increased ABA contents correlate with reduced ROS production and with enhanced disease progression.

### Manipulation of ABA Level and *Dickeya dadantii* Virulence

Using ABA-deficient or overproducing plants, we have demonstrated that *Arabidopsis* resistance to the soft rot bacterium *D. dadantii* is negatively correlated with the accumulation of ABA (**Figure 1**). Moreover, ABA contents increased in WT plants in response to inoculation (**Figure 2**), probably establishing favorable physiological conditions for disease progression. We detected this increase only after infiltration of leaves with quite a high inoculum density (10^7^ cfu ml^-1^) (**Figure 2**) that introduces many bacterial cells into a broad leaf volume and not with a lower inoculum density (10^5^ cfu ml^-1^) or when using the immersion method that allows penetration of few bacteria at discrete sites. A similar dependence of increases in ABA levels with infiltrated inoculum concentration has also been observed for interactions between *P. syringae* and *Arabidopsis* using equivalent bacterial concentrations (de Torres-Zabala et al., 2007). This dose-response relationship may indicate that the degree of ABA accumulation is closely linked to the intensity of the interactions between bacterial and plant cells. Nonetheless, the increase in the expression of ABA biosynthesis genes occurs early after plant immersion in a bacterial suspension (**Figure 3**). The accumulation of transcripts for ABA biosynthesis genes may trigger an increase in ABA content that would modify leaf physiology and favor disease development.

Several studies described an enhanced production of ABA upon infection by necrotrophic fungi as *B. cinerea*, *Plectosphaerella cucumerina* (Garcia-Andrade et al., 2011) and *Alternaria brassicicola* (Mazumder et al., 2013). In these cases, ABA production participates to the reinforcement of plant resistance, potentiating callose deposition (Garcia-Andrade et al., 2011) or inducing JA-related defense pathways in the resistant plant *Sinapis alba* (Mazumder et al., 2013). But ABA could also regulate negatively plant resistance to *P. cucumerina*, inhibiting SA, JA, or ET-related defense pathways (Sánchez-Vallet et al., 2012). Nevertheless, pathogenic bacteria exhibit an intercellular growth phase in plant tissues and should be considered as hemibiotrophs (Kraepiel and Barny, 2016), which makes it difficult to compare fungal and bacterial rotting pathogens. Manipulation of ABA homeostasis by bacterial pathogens has also been described in pathosystems involving T3SS-dependent bacteria and this proved to be an important process favoring virulence of *P. syringae* pv. *tomato* and *Xanthomonas campestris* pv. *campestris* (de Torres-Zabala et al., 2007; Ho et al., 2013). Enhancement of ABA biosynthesis in infected tissues would appear to be a common mechanism, although genes targeted may differ. Type III effectors of the T3SS-dependent bacteria induce the expression of *NCED* genes. In contrast, *D. dadantii* infection increased transcript abundance for *AAO3* and *ABA3*, both involved in the production of a functional ABA-aldehyde oxidase catalyzing the last step of ABA biosynthesis. *D. dadantii* virulence relies essentially on cell wall degrading enzymes secreted into plant tissues through a T2SS. We showed that the *D. dadantii hrcC* type III secretion mutant, whose virulence is not significantly affected in our conditions (Lebeau et al., 2008), induces *AAO3* and *ABA3* genes in a similar manner to the WT strain. In contrast, infection by the *prtE* or *outC* mutants, respectively defective in the T1SS and T2SS, triggered at best only a weak induction of the expression of both genes (**Figure 3**). Enzymes secreted by these two systems, or the secretion systems themselves, are thus required to produce a signal that stimulates the expression of ABA biosynthesis genes. Proteolytic activation of cell wall degrading enzymes could participate in this cooperation of both secretion systems (Shevchik et al., 1998; Creze et al., 2008). Further analysis of the ability of different bacterial mutants to induce ABA biosynthesis is required to test this hypothesis.

### A Central Place for ABA in the Interaction between *Arabidopsis* and *Dickeya dadantii*

To determine the role of ABA-status in the interaction between *Arabidopsis* and *D. dadantii*, we also examined the expression of bacterial virulence factors encoding genes during infection of plants with modified ABA contents. Induction of the expression of three bacterial genes, namely *indC*, *pelI* and *prtC*, was lower at 24 and 30 hpi for infected ABA-deficient *aba3-1* plants compared to WT. In contrast, no significant effect was observed on bacterial gene expression from ABA overproduction in *35S::NCED6* plants. We could thus hypothesize that in infected WT plants the induction of ABA biosynthesis was already at a sufficiently high threshold for full induction of bacterial virulence.

Interestingly, among the virulence genes differentially expressed during *aba3-1* and Col-0 infection, were *prtC* and *pelI* genes that encode proteins secreted through the type I and the type II secretion systems, respectively, and these secretion systems are both required for increased expression of ABA biosynthesis genes (**Figures 3**, **4**). It is thus tempting to speculate that a positive feedback exists during infection between ABA biosynthesis and the production of bacterial enzymes. Interestingly, ABA mutants have been shown to exhibit modified cell wall composition and structure in tomato and *Arabidopsis*. The *aba1-6* mutant of *Arabidopsis* has lower cellulose content and more cell wall-associated uronic acid than the corresponding WT (Sánchez-Vallet et al., 2012) whereas the *sitiens* mutant of tomato exhibits, mainly, a higher degree of pectin methylation (Curvers et al., 2010). The most important *D. dadantii* pectate lyases (Pel) are enzymes that degrade mainly demethylated pectins (Barras et al., 1994; Abbott and Boraston, 2008) and the modifications of cell wall composition observed in the ABA mutants could lead to an increased resistance to bacterial cell wall degrading enzymes. Furthermore, the chemical structure of the resulting oligosaccharides could also differ between the ABA mutant and the WT, as observed in tomato (Curvers et al., 2010), leading to a different signaling activity. Moreover, ABA mutants exhibit alteration of their cuticle integrity associated to higher resistance to pathogens (Curvers et al., 2010; L’Haridon et al., 2011; Survila et al., 2016). A defective cuticle could allow a faster defense reaction to pathogen elicitors but also constitutively activate defense mechanisms. L’Haridon et al. (2011) demonstrated that alteration of the cuticle integrity, by wounding or by mutations, leads to ROS production and resistance to *B. cinerea*. Recently, Survila et al. (2016) showed that class III peroxidase activity enhances ROS production and loss of cuticle integrity. Interestingly, ABA inhibits this oxidative stress and compromises resistance, whereas ABA biosynthetic mutants exhibit an increased cuticle permeability associated to high ROS production and increased resistance to *B. cinerea* and *Pectobacterium carotovorum*.

Induction of the indigoidine biosynthesis gene *indC* expression is also suppressed in infected *aba3-1* plants. Indigoidine biosynthesis allows *D. dadantii* to counter the oxidative burst produced by infected plant tissues (Reverchon et al., 2002). *indC* expression is only weakly induced by ROS in *in vitro*-cultured cells, but highly activated *in planta* (Reverchon et al., 2002; Chapelle et al., 2015). Furthermore, the lack of induction of *indC* expression on infection of *aba3-1* plants is associated with a stronger H_2_O_2_ production than in the WT (**Figures 4**, **6**). This indicates that the strong induction of *indC* expression observed *in planta* could be linked to ABA induced modifications, and unrelated to the oxidative stress. This is in accordance with previous results showing that the expression of indigoidine biosynthesis genes is regulated by several master regulators *in planta* in response to multiple signals (Mhedbi-Hajri et al., 2011; Reverchon and Nasser, 2013).

### Peroxidase Activity, Oxidative Stress, and ABA-Induced Susceptibility to *Dickeya dadantii*

Analysis of plant susceptibility to *D. dadantii* and DAB-staining performed in this work confirm the strong correlation between resistance and H_2_O_2_ production in leaves (**Figures 5**, **6**; Fagard et al., 2007; Kraepiel et al., 2011). AtRBOHD activity was shown to be the principal effector for the rapid production of ROS in plant defense, as illustrated by the dramatic increase of susceptibility of the *AtrbohD* mutant (**Figure 5**; Fagard et al., 2007). In contrast, *D. dadantii* infection of an *aba3-1*-*AtrbohD* double mutant resulted in intermediate susceptibility phenotypes and oxidative stress, indicating that infection activates a second source of ROS, independent of AtRBOHD activity. ABA inhibits this second ROS producing mechanism, since it was only observed in an ABA-deficient genotype. This ABA-induced weakening of ROS production should be part of a larger defensive network where ABA acts as a moderating signal. Indeed, transcriptomic and epigenetic studies revealed that ABA down-regulates the expression of many defensive genes (de Torres-Zabala et al., 2007; Cheng et al., 2016; Liao et al., 2016). All these additive effects would explain the extremely susceptible phenotype of the ABA-overproducing plants, even more pronounced than the *AtrbohD* mutant’s one even if this mutant does not exhibit any oxidative stress response to infection (**Figures 5**, **6**).

Class III peroxidases are glycoproteins located in vacuoles and cell walls that could catalyze the formation of the superoxide radical ion, which undergoes dismutation to H_2_O_2_, as part of plant defense (Almagro et al., 2009). Total class III peroxidase activity can be measured spectrophotometrically *in vitro* using the artificial phenolic substrate guaiacol regardless of *in vivo* substrate preference (Cesarino et al., 2012). In this study, we detected much higher peroxidase activity in genotypes containing the *aba3-1* mutation, namely in *aba3-1* compared to WT and *aba3-1-AtrbohD* compared to *AtrbohD* (**Figure 7**). This inhibition of peroxidase activity by ABA could represent a major control point in plant stress physiology since Ghassemian et al. (2008) showed that, among the 73 members of the large class III peroxidase gene family in *Arabidopsis* (Welinder et al., 2002), 14 are down-regulated by ABA at the transcriptional level, and Sánchez-Vallet et al. (2012) reported the up-regulation of the peroxidase-encoding gene *PRX33* in the *aba1-6* mutant of *Arabidopsis*.

Our results suggest that H_2_O_2_ accumulation in infected plants that are ABA-deficient could be linked to their high peroxidase activity. In all the genotypes tested, however, infection with the WT *D. dadantii* strain 3937 failed to increase peroxidase activity *in vitro* compared to buffer-inoculation, even though the oxidative burst was specific to bacteria-infected leaves (**Figures 6**, **7**). The peroxidase activity we quantified *in vitro* after protein extraction reflected the amount of class III peroxidases present in the leaf tissues irrespective of their effective activity *in vivo*. According to Allan and Fluhr (1997), we can hypothesize that peroxidase substrates are generated specifically in response to infection, triggering H_2_O_2_ production from peroxidases accumulated in ABA-deficient plants. Plant metabolic responses to *D. dadantii* infection, to activity of bacterial cell wall degrading enzymes or to bacterial metabolites could constitute a source of class III peroxidase substrates. Metabolomic studies would be useful for the identification of potential peroxidase substrates. Furthermore, the pH optima for class III peroxidase activities are neutral to basic and they are mostly inactive in unstressed acidic cell walls (Bolwell and Wojtaszek, 1997). During the initial stages of *D. dadantii* infection, bacteria induce an alkalization of the cell walls, which in turn induces the activity of the bacterial transcriptional activator MfbR, the synthesis of cell wall degrading enzymes and their activity (Reverchon et al., 2010). The alkalization of the cell wall by infection could also activate cell wall-localized plant peroxidases and specifically initiate H_2_O_2_ production in response to the presence of bacteria. We cannot exclude that ROS overproduction in ABA deficient mutants is not a result of AtRBOHD or peroxidase activity, but a third unidentified enzymatic process. Indeed, RBOHF as been shown to exhibit a specific spatio-temporal pattern of expression upon infection by *P. cucumerina* and *P. syringae* (Morales et al., 2016) and different types of oxidases have been implicated in the oxidative stress encountered after pathogen infection (Apel and Hirt, 2004). Our results are nonetheless consistent with the reported role of peroxidases in ROS production after infection of tomato by *D. dadantii* (Asselbergh et al., 2008a) and with the observation that the levels of constitutively produced peroxidases are decisive in ROS production (Bolwell and Wojtaszek, 1997).

### A Likely Direct Effect of ABA on Susceptibility

Abscisic acid increases plant susceptibility to many bacterial and fungal pathogens interacting with the SA-dependent pathway (Audenaert et al., 2002; Cao et al., 2011; Xu et al., 2013) or with JA and ET-dependent pathways (Anderson et al., 2004; Fan et al., 2009). Our work strongly suggests that ABA could also play a direct negative effect in a basal constitutive defense mechanism. Class III peroxidase activity depends on ABA production (**Figure 7**) and could contribute to the ability of plant tissues to produce H_2_O_2_ rapidly in response to pathogen attack (**Figure 6**). In *Arabidopsis*, SA-related responses are inefficient against *D. dadantii* (Fagard et al., 2007), while ROS production and JA signaling represent effective defense reactions (Fagard et al., 2007; Kraepiel et al., 2011). The latter plant responses to *D. dadantii* are unrelated since the JA insensitive mutant *coi1* and the JA deficient mutant *jar1* produce H_2_O_2_ at levels comparable to those of WT in response to infection (Supplementary Figure S2). It is thus likely that the ABA-related control of H_2_O_2_ accumulation does not involve JA signaling. Infection-triggered ROS accumulation would thus be a new direct effect of ABA in plant defense, besides the ones previously described in other pathosystems, such as phenylalanine ammonia lyase production, callose deposition, stomatal closure or involvement in the priming of defense (Asselbergh et al., 2008b; Ton et al., 2009; Garcia-Andrade et al., 2011; Pastor et al., 2013).

In conclusion, we demonstrate here that ABA content in *Arabidopsis* leaves is a strong determinant of *D. dadantii* virulence. Our results reinforce that ABA plays a pivotal role during the plant–bacteria interaction through the stimulation of virulence factor expression in invading bacteria and the inhibition of ROS production in plant tissues. This latter effect of ABA appears to result from its negative control of the production of class III peroxidases. The opposite results obtained using ABA deficient or ABA overproducing plants support our conclusions. Thus, manipulation of ABA homeostasis in plants, through the up-regulation of genes involved in the last step of ABA-biosynthesis, is part of the virulence strategy of *D. dadantii*. Interestingly, ABA would appear to modulate a sentinel process that participates in the basal defense, consisting of the production of a threshold level of peroxidases that are specifically activated in response to pathogen attack.

## Author Contributions

FVG, JP, and YK designed the research, interpreted the data and wrote the manuscript. YK, OP, RG, ES-C, AM-G, and PP performed experiments and analyzed data. LB performed statistical analysis for **Figure 3**.

## Conflict of Interest Statement

The authors declare that the research was conducted in the absence of any commercial or financial relationships that could be construed as a potential conflict of interest.

## Footnotes

1 http://www.waters.com
