# Supplementary material for: Manipulation of ABA Content in Arabidopsis thaliana Modifies Sensitivity and Oxidative Stress Response to Dickeya dadantii and Influences Peroxidase Activity
Source: Front Plant Sci. 2017 Apr 3;8:456. doi: 10.3389/fpls.2017.00456 (PMC5376553; doi:10.3389/fpls.2017.00456)
Supplement: Supplementary file 1 [file Table_1.PDF]

Supplementary Table 1. Statistical analysis of symptom progression on the Col-0 wild type, the *aba3-1* ABA deficient mutant, the *35S::NCED6* ABA overproducing plant, the *rbohD* mutant and the *rbohD-aba3-1* double mutant. All the pair-wise comparisons have been performed using the Fisher's exact test (two sided p-value). The p-values correspond to the pair-wise comparison of frequencies of stage 0 and stages ≥ 2 at two dpi and of stage 3 at seven dpi. ***, p < 0,001; **, p < 0,01 *, p < 0,05.

	p-values 2 dpi, stage 0					p-values 2 dpi, stage ≥2					p-values 2 dpi, stage 3				
	WT	*aba3-1*	*35S :: NCED6*	*rbohD*	*rbohD-aba3-1*	WT	*aba3-1*	*35S :: NCED6*	*rbohD*	*rbohD-aba3-1*	WT	*aba3-1*	*35S :: NCED6*	*rbohD*	*rbohD-aba3-1*
WT	1	**	*	***	***	1	***	***	**	0.13	1	***	***	***	0.16
aba3-1		1	***	***	***		1	***	***	*		1	***	***	***
35S :: NCED6			1	*	0.47			1	0.38	***			1	**	***
rbohD				1	0.12				1	***				1	***
rbohD-aba3-1					1					1					1
