# Supplementary material for: Manipulation of ABA Content in Arabidopsis thaliana Modifies Sensitivity and Oxidative Stress Response to Dickeya dadantii and Influences Peroxidase Activity
Source: Front Plant Sci. 2017 Apr 3;8:456. doi: 10.3389/fpls.2017.00456 (PMC5376553; doi:10.3389/fpls.2017.00456)
Supplement: Supplementary file 2 [file Table_2.PDF]

Supplementary Table 2. Statistical analysis of expression of the ABA biosynthetic genes *AAO3* and *ABA3* following the infection by the 3937 wild type bacterial strain and the *outC*, *prtE* and *hrcC* secretion mutants. Each kinetic is compared to the buffer using a linear mixed-effects model with the bacterial strain as random effect (R software 3.3.1). DF, degree of freedom.

	Fixed effect: strain * hours post inoculation					
	AAO3			*ABA3*		
strains	DF	t-value	p-value	DF	t-value	p-value
3937	25	2.7239	0.0116	25	4.1336	0.0004
prtE	25	0.5740	0.5711	25	1.8058	0.0830
outC	25	0.6054	0.5504	25	2.1536	0.0411
hrcC	25	2.4050	0.0239	25	3.9452	0.0006
