# Supplementary material for: Manipulation of ABA Content in Arabidopsis thaliana Modifies Sensitivity and Oxidative Stress Response to Dickeya dadantii and Influences Peroxidase Activity
Source: Front Plant Sci. 2017 Apr 3;8:456. doi: 10.3389/fpls.2017.00456 (PMC5376553; doi:10.3389/fpls.2017.00456)
Supplement: Supplementary file 3 [file Image_1.PDF]

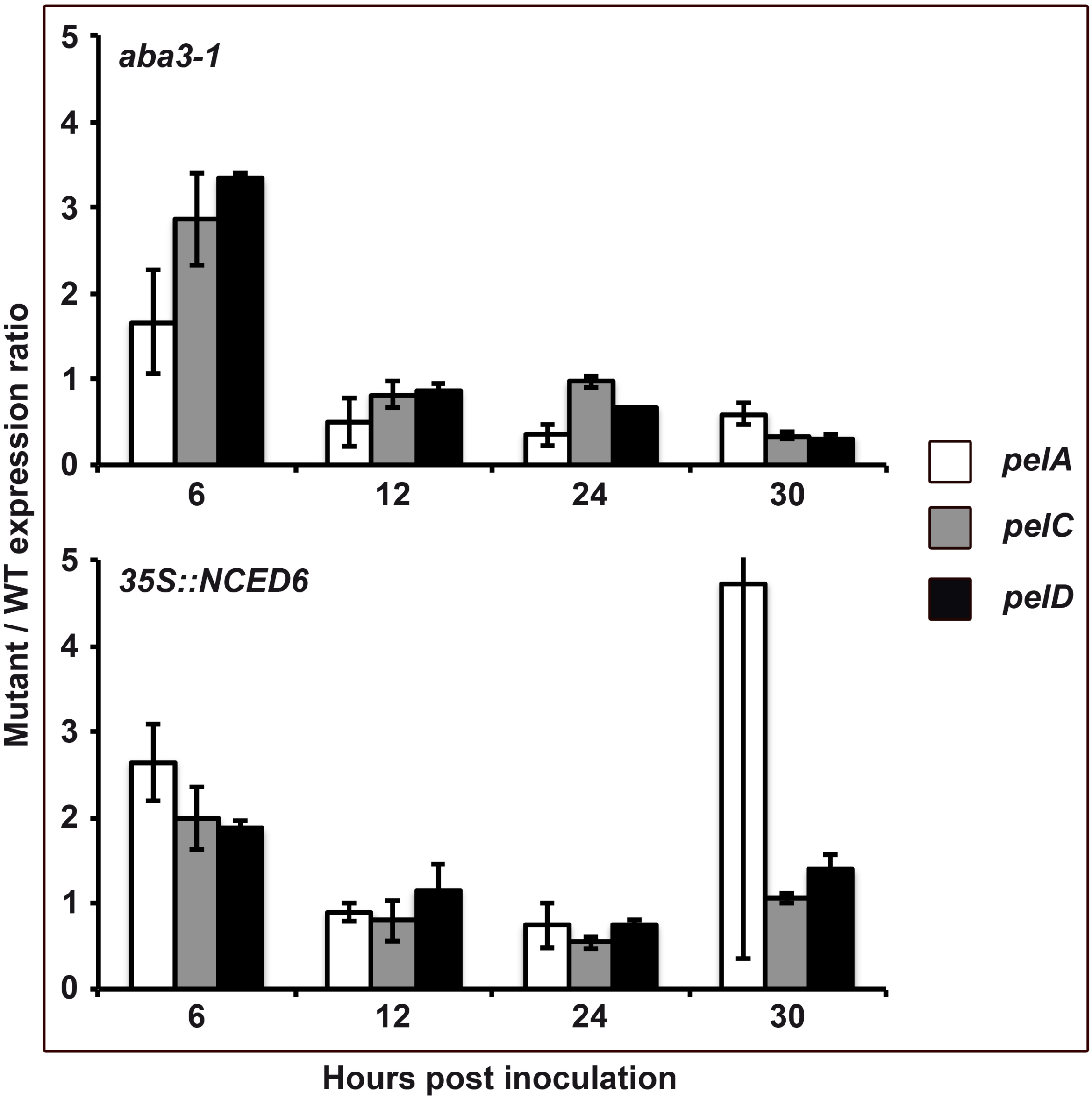

Supplementary Figure 1. Effect of plant ABA content on *pelA*, *pelC* and *pelD* genes expression. The 3937 bacterial wild type strain was inoculated by immersion of wild type Col-0, the *aba3-1* mutant and *35S::NCED6* plants. Four to six plant rosettes were harvested at the indicated time points and expression of bacterial genes encoding the pectinases PelA, PelC and PelD were analyzed by quantitative real time RT-PCR using *RpoB* as a constitutive gene. Transcript accumulation was quantified after infection of *aba3-1* and *35S::NCED6* plants and was expressed as the ratio of mutant or transgenic expression to wild type (WT) (mutant / WT expression ratio), comparing levels measured on genotypes at the same time point after inoculation. Bars correspond to the mean of 2 (*pelC* and *pelD)*) or 3 (*pelA*) replicates and the error bars represent standard deviation. Given that expression ratios for the three genes were a little less than 0.5 for only one time point, differences between WT and mutants were not considered clear enough to be biologically relevant.
