# Supplementary material for: Manipulation of ABA Content in Arabidopsis thaliana Modifies Sensitivity and Oxidative Stress Response to Dickeya dadantii and Influences Peroxidase Activity
Source: Front Plant Sci. 2017 Apr 3;8:456. doi: 10.3389/fpls.2017.00456 (PMC5376553; doi:10.3389/fpls.2017.00456)
Supplement: Supplementary file 4 [file Image_2.PDF]

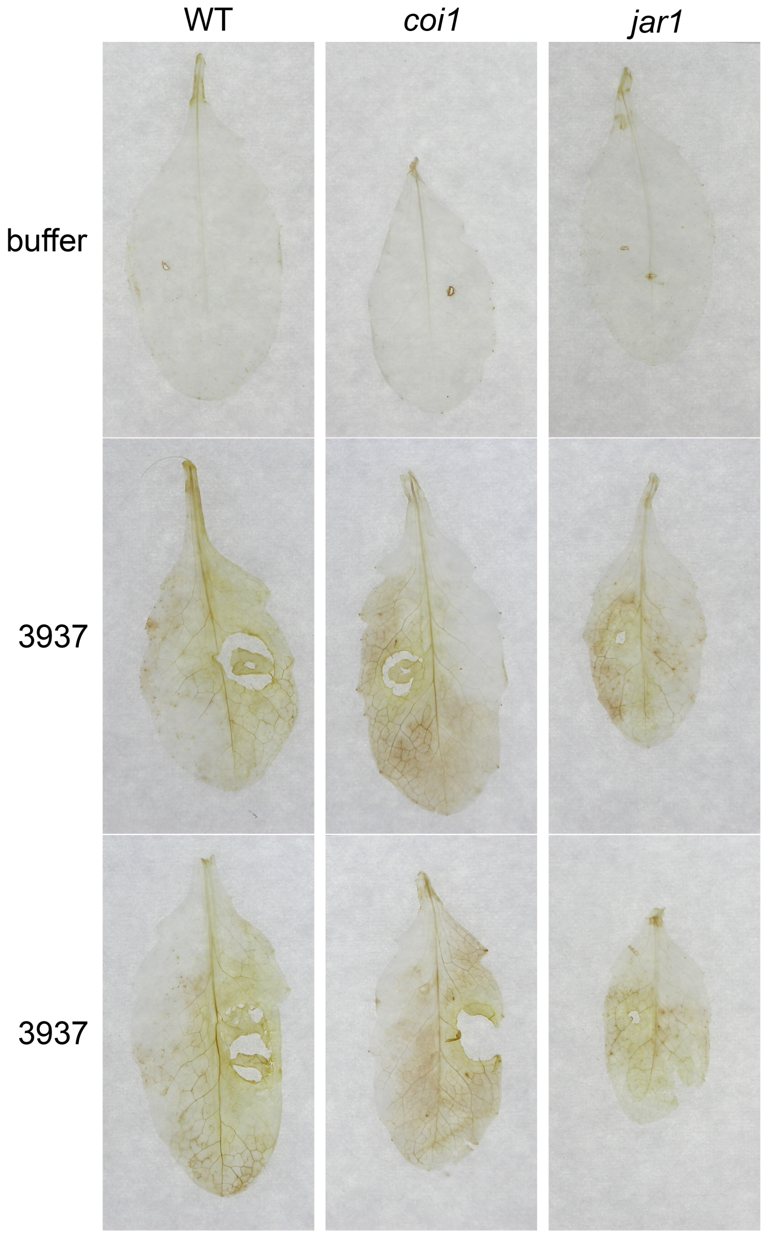

Supplementary Figure 2. Diaminobenzidine-staining of H_2O_2 produced in leaves of the wild type Col-0 and jasmonate-related mutants infected with *Dickeya dadantii*. *coi1* is a JA insensitive mutant (Ellis and Turner, 2002, *Planta* 215: 549-556) and *jar1* is impaired in the biosynthesis of the biological active JA-Ile conjugate (Staswick *et al.*, 2002, *Plant Cell* 14: 1405-1415). Infection was performed by depositing 5 µL of a 5.10^7 cfu/mL wild type bacterial strain (3937) suspension onto a needle-wound. Twenty-four hours post-inoculation, leaves exhibiting a stage 1 symptom (see methods and figure 1) were selected for staining. The experiment was performed three times with similar results and 2 representative leaves are presented from at least 12 analysed per experiment. Buffer inoculation was used as control.
